# The burden of healthcare-associated infections in Brazil: multi-hospital point prevalence using a matched case-control study

**DOI:** 10.1590/1516-3180.2023.0307.R1.03072024

**Published:** 2025-04-28

**Authors:** Luiz Gustavo Machado, Daiane Silva Resende, Paola Amaral de Campos, Iara Rossi, Melina Lorraine Ferreira, Iolanda Alves Braga, Caio Augusto Martins Aires, Maria Tereza Freitas Tenório, Paulo Pinto Gontijo-Filho, Sabrina Royer, Rosineide Marques Ribas

**Affiliations:** I Laboratory of Molecular Microbiology, Universidade Federal de Uberlândia (UFU), Uberlândia (MG), Brazil.; II Laboratory of Molecular Microbiology, Universidade Federal de Uberlândia (UFU), Uberlândia (MG), Brazil.; III Laboratory of Molecular Microbiology, Universidade Federal de Uberlândia (UFU), Uberlândia (MG), Brazil.; IV Laboratory of Molecular Microbiology, Universidade Federal de Uberlândia (UFU), Uberlândia (MG), Brazil.; V Laboratory of Molecular Microbiology, Universidade Federal de Uberlândia (UFU), Uberlândia (MG), Brazil.; VI Laboratory of Molecular Microbiology, Universidade Federal de Uberlândia (UFU), Uberlândia (MG), Brazil.; VII Health Science Department, Universidade Federal Rural do Semi-Árido (UFERSA), Mossoró (RN), Brazil.; VIII Hospital Infection Control Service, Santa Casa de Misericórdia de Maceió (AL), Brazil.; IX Laboratory of Molecular Microbiology, Universidade Federal de Uberlândia (UFU), Uberlândia (MG), Brazil.; X Laboratory of Molecular Microbiology, Universidade Federal de Uberlândia (UFU), Uberlândia (MG), Brazil.; XI Laboratory of Molecular Microbiology, Universidade Federal de Uberlândia (UFU), Uberlândia (MG), Brazil.

**Keywords:** Infection control, Multicenter study, Intensive care units, Epidemiology, Healthcare-associated infections, Point prevalence, Infections, High use of antibiotics

## Abstract

**Background:**

Healthcare-associated infections (HAIs) have a significant impact on patient care worldwide and have serious implications for the Brazilian healthcare system.

**Objectives:**

This study aimed to describe the trends in HAIs in adult intensive care units (ICUs) using data from a national point-prevalence survey.

**DESIGN AND SETTING:**

A point-prevalence study was conducted in 2019 across adult intensive ICUs in large acute care hospitals in Brazil.

**METHODS:**

A matched case-control study was performed to assess the risk factors associated with the development of infection.

**RESULTS:**

A total of 386 patients from 15 hospitals were studied, of whom 102 (26.4%; 102/386) were infected, and 76.5% had at least one ICU-acquired infection. In clinical-surgical ICUs (CSU), the prevalence of infections acquired in the unit was 77.9%, whereas in Coronary ICUs (COU), it was 68.7%. There was a predominance of pneumonia (51.0%), mainly caused by Gram-negative non-fermenters, and bloodstream infections (34.4%), predominantly caused by coagulase-negative ***Staphylococcus*** (CoNS). In the risk factor analysis, cancer and general antimicrobial use were independently associated.

**CONCLUSION:**

This study found a high burden of HAIs in adult ICUs in Brazil, mainly associated with the high use of antibiotics for infections and a worse prognosis.

## INTRODUCTION

Healthcare-associated infections (HAIs) and antimicrobial resistance are growing global public health concerns, with particular significance in intensive care units (ICUs).^1^ In Brazilian ICUs, estimates of HAI prevalence in tertiary hospitals range from 44.3% to 79.4%.^2,3,4^

Comprehensive data on infection types, pathogenic microorganisms, risk factors, and antimicrobial use across Brazil are essential for developing policies that prioritize the prevention and treatment of HAIs. Such data can help optimize patient care and efficiently allocate financial resources.^5,6^ In 2016, a multicenter study was conducted in Brazil to determine the one-day point prevalence of infections in 28 ICUs in the state of Minas Gerais. The study included 303 patients, of whom 155 (51.2%) were found to be infected, and 123 (79.4%) had at least one infection acquired in the ICU.^4^

The current study was conducted in 2019 using a design similar to that previously described but including additional Brazilian states. We hypothesized that the prevalence of HAIs and associated microorganisms would vary significantly among geographical regions. To gain a broad understanding of the magnitude of HAIs in ICUs and the associated burden of disease in Brazil, we estimated the prevalence of HAIs using data from multicenter studies.

## OBJECTIVE

This article aimed to describe trends in HAIs in adult intensive care units (ICUs) using data from a national point-prevalence survey.

## METHODS

### Survey design and participating hospitals

This matched case-control study was conducted in 17 clinical-surgical ICUs (CSUs) and five coronary ICUs (COUs) across the five main geographic regions of Brazil (North, Northeast, Midwest, Southeast, and South) in 2019. Fifteen hospitals, comprising both public and private institutions, participated in the study with the consent of their administrations. This study included 386 patients hospitalized in the ICUs on the corresponding day. The prevalence of HAIs and episodes of infection were determined for each region, along with the overall frequency of microorganisms diagnosed in each type of ICU. For the microorganisms evaluated in this study, data were provided by the respective hospitals at the time of microbiological diagnosis. This case-control study was based on a one-day point prevalence survey to determine the demographic characteristics and risk factors between groups. The analyzed variables included age, length of hospital stay, underlying diseases, hospital risk factors, and antimicrobial therapy. This study was approved on August 25, 2018, by the Research Ethics Committee of the Federal University of Uberlandia under the protocol number CAAE: 88387817.0.0000.5152.

The co-participating centers were randomly selected. A survey of hospitals with ICU beds was conducted in the main cities of each region, and the managers of these institutions were contacted to complete the project. The executing team applied the same methodology to all centers for data collection and surveys in the visited ICUs.

### Definitions

HAIs were defined according to the guidelines of the Agência Nacional de Vigilância Sanitária (ANVISA, Brazil),^7^ which were largely based on definitions from the National Healthcare Safety Network (NHSN).^8^ However, ANVISA’s guidelines expand the definition of bloodstream infections (BSIs) to include patients with clinically defined sepsis without laboratory confirmation. Treatment administered during the period between the suspicion of infection and obtaining susceptibility results was defined as empirical.^9^

### Selection of cases

Cases were defined as patients who had a confirmed HAI acquired in the ICU up to the corresponding day in each hospital, as defined by physicians according to the Diagnostic Criteria for Infection Related to Health Care established in each institution, according to ANVISA. At least one case was selected from each ICU included in the prevalence survey. Patient pairing was performed in a 1:1 ratio for patients who met the established criteria.

### Selection of controls

Controls were patients without HAI who met the following predetermined criteria: they needed to be hospitalized in the same unit as the patient and should not have acquired an infection until the corresponding day. Controls were matched according to sex, age, reason for hospitalization (clinical, surgical, or traumatic), and the total length of hospital stay before infection (risk time).

To eliminate time bias, the total hospitalization time for controls until the corresponding day should be greater than or equal to the interval between the admission and infection dates of the cases.^10^ In addition, controls had to be in the same age group as the case patients, with a maximum age difference of ± 10 years.

### Statistical analysis

Comparisons between groups were made using Student’s t-test for variables with a normal distribution (evaluated using the D’Agostino and Lilliefors tests) and the Mann-Whitney U test for variables with a non-normal distribution. Chi-squared and Fisher’s exact tests were used to assess the relationships between categorical variables. Multiple regression models were used for multivariate analysis. A significance level of 5% (P < 0.05) was considered significant. All analyses were performed using BioEstat 5.0 software (Instituto de Desenvolvimento Sustentável Mamirauá, Tefé, AM, Brazil).

## RESULTS

This study included 15 hospitals located in the five regions of Brazil. The institutions had 4,204 beds, of which 337 (8.0%) were CSUs and 99 (2.3%) were COUs. **Table 1** shows the prevalence of HAIs in ICUs on the day of the study. Among the CSUs, 86/300 (28.7%) patients had HAIs, and 77.9% of these infections were acquired within the unit. In addition, when the frequency was examined by region, the Southwestern (39.7%) and Northern (35.5%) regions had the highest rates. Regarding the prevalence in the COUs, 16/86 (18.6%) patients had at least one infectious episode, and 68.7% of these infections were acquired in the unit.

**Table 1:** Prevalence of healthcare-associated infections in adult clinical-surgical and coronary intensive care units in different regions in Brazil

Regions	Number of Hospitals	Patients admitted to the ICU^*^		Patients with HAI^§^(%)		Patients with HAI acquired in the ICU (%)	
		CSU^¥^	COU^◆^	CSU	COU	CSU	COU
North	3	45	13	16 (35.5)	5 (38.5)	13 (81.2)	4 (80.0)
Northeast	5	99	24	19 (19.2)	3 (12.5)	13 (68.4)	2 (66.7)
Midwest	2	60	0	14 (23.3)	0	13 (92.8)	0
Southwest	4	83	49	33 (39.7)	8 (16.3)	24 (72.7)	5 (62.5)
South	1	13	0	4 (30.8)	0	4 (100.0)	0
Total	15	300	86	86 (28.7)	16 (18.6)	67 (77.9)	11 (68.7)

^*^ICU = intensive care unit;

^§^HAI = healthcare-associated infection;

^¥^CSU = clinical-surgical unit;

^◆^COU = coronary unit.

**Table 2** shows the frequency of infectious episodes identified according to the site of infection in HAIs acquired exclusively in the ICUs. A total of 84 episodes were identified in 67 infected patients in CSUs, with 12 episodes observed in 11 infected patients in COUs. Pneumonia episodes were the most prevalent infections in both units, accounting for 50.0% of CSUs and 58.3% of COUs, followed by BSIs (34.5% and 33.3 %, respectively). The frequency of episodes by region followed the same pattern, except in the southeastern region, where pneumonia and BSIs had the same frequency.

**Table 2:** Prevalence of healthcare-associated infection episodes in adult clinical-surgical and coronary intensive care units in different regions of Brazil

Regions	Total number of HAI^*^ episodes acquired in the ICU		Pneumonia (%)		Bloodstream infection (%)		Urinary tract infection (%)	
	CSU^§^	COU^¥^	CSU	COU	CSU	COU	CSU	COU
North	17	5	8 (47.0)	4 (80.0)	6 (35.3)	0	2 (11.8)	0
Northeast	17	2	8 (47.0)	0	7 (41.2)	2 (100.0)	2 (11.8)	0
Midwest	14	0	9 (64.3)	0	5 (35.7)	0	0	0
Southwest	26	5	10 (38.5)	3 (60.0)	10 (38.5)	2 (40.0)	4 (15.4)	0
South	10	0	7 (70.0)	0	1 (10.0)	0	1	0
Total	84	12	42 (50.0)	7 (58.3)	29 (34.5)	4 (33.3)	9 (10.7)	0

^*^HAI = healthcare-associated infection;

^§^CSU = clinical-surgical unit;

^¥^COU = coronary unit; Other infections – CSU = surgical site (2); skin (1).

The microorganisms responsible for HAIs are shown in **Table 3**. A total of 45 microorganisms were identified in 96 episodes (46.9%) of HAI acquired in the ICUs. The most common microorganisms were Gram-negative bacilli, accounting for 34.4% (n = 33/96 episodes) of the total documented pathogens. In CSUs, the most common bacteria were *Pseudomonas aeruginosa*, Coagulase-Negative *Staphylococcus* (CoNS), and *Klebsiella pneumoniae*, all having the same proportions (18.9%), followed by *Acinetobacter baumannii* (13.5%) and *Enterobacter cloacae* (10.8%). The most common agents causing pneumonia in these units were *A. baumannii* (22.2%) and *P. aeruginosa* (22.2%). In BSIs, the most prevalent microorganism was CoNS, accounting for approximately half of all cases. Furthermore, we observed a low frequency (48.8%; data not provided) of infections based on the microbiological diagnostic criteria.

**Table 3:** Microorganism frequency by common healthcare-associated infections acquired in adult clinical-surgical and coronary intensive care units in Brazil

	Total HAI^*^	Bloodstream infection	Pneumonia
**Clinical-surgical ICU^§^**			
Patients with HAI; N	67	29	40
Number of microorganisms identified; n	41	14	21
Microorganisms (N; %)	*Pseudomonas aeruginosa* (7; 18.9%)	CoNS^¥^(6; 42.8%)	*Acinetobacter baumannii* (4; 22.2%)
	CoNS^¥^ (7; 18.9%)	*Pseudomonas aeruginosa* (2; 14.3%)	*Pseudomonas aeruginosa* (4; 22.2%)
	*Klebsiella pneumoniae* (7; 18.9%)	*Klebsiella pneumoniae* (2; 14.3%)	*Klebsiella pneumoniae* (3; 16.7%)
	*Acinetobacter baumannii* (5; 13.5%)	*Enterobacter cloacae* (1; 7.1%)	*Enterobacter cloacae* (2; 11.1%)
	Other Gram-negative bacilli^◆^ (5; 13.5%)	*Klebsiella oxytoca* (1; 7.1%)	*Staphylococcus aureus* (2; 11.1%)
	*Enterobacter cloacae* (4; 10.8%)	*Pseudomonas stutzeri* (1; 7.1%)	CoNS^¥^(1; 5.5%)
	*Escherichia coli* (2; 5.4%)	*Escherichia coli* (1; 7.1%)	*Proteus mirabilis* (1; 5.5%)
	*Staphylococcus aureus* (2; 5.4%)		*Raoultella ornithinolytica* (1; 5.5%)
	*Candida albicans* (1; 2.7%)		*Citrobacter freundii* complex (1; 5.5%)
	*Streptococcus pneumoniae* (1; 2.7%)		*Candida tropicalis* (1; 5.5%)
			*Streptococcus pneumoniae* (1; 5.5%)
**Coronary ICU**			
Patients with HAI^*^; N	11	6	8
Number of microorganisms identified; n	4	2	2
Microorganisms (frequency)	*Klebsiella pneumoniae* (2; 50.0%)	*Klebsiella pneumoniae* (1; 50.0%)	*Klebsiella pneumoniae* (1; 50.0%)
	*Staphylococcus aureus* (1; 25.0%)	*Acinetobacter baumannii* (1; 50.0%)	*Staphylococcus aureus* (1; 50.0%)
	*Acinetobacter baumannii* (1; 25.0%)		

^*^HAI = healthcare-associated infection;

^§^ICU = intensive care unit;

^¥^CoNS = Coagulase-Negative *Staphylococcus: Staphylococcus epidermidis* (3), *Staphylococcus hominis* (2), *Staphylococcus haemolyticus* (1), *Staphylococcus capitis* (1);

^◆^ *Pseudomonas stutzeri* (1), *Klebsiella oxytoca* (1)*, Proteus mirabilis* (1), *Raoultella ornithinolytica* (1), *Citrobacter freundii* complex (1)*.*

Risk factor analysis was performed using a matched case-control study, and 50 pairs were selected for analysis. The matching criteria were selected to ensure that there were no significant differences between the groups. Success rates ranged from 56.0% to 86.0% (**Table 4**).

**Table 4:** Success rate of paired variables for risk factors in patients with healthcare-related infections through a case-control study paired in intensive care units in Brazil

Variables	Total of pairs; n	No. of pairs reached; n	Success achieved (%)	P value
Reason for hospitalization	50	41	82.0	0.3284^*^
Sex	50	43	86.0	0.6787^*^
Age	50	29	58.0	0.4969^◆^
Risk time	50	28	56.0	0.6439^§^

^*^Chi-square test;

^§^Student’s t-test;

^◆^Mann-Whitney test.

In general, the patients were relatively young, with 57.0% younger than 65 years, and the majority (78.3%) used three or more invasive devices. Approximately 59.3% of the patients were treated with broad-spectrum antibiotics, such as β-lactams with inhibitors (24.1%) and carbapenems (19.0%) (data not shown). Among all patients, 59.8% used antimicrobials, whereas only 24% of the control patients received such treatment. **Table 5** compares the cases and controls, revealing significant differences between patients with cancer and those using a central venous catheter, mechanical ventilation, tracheostomy, and enteral nutrition. Patients who used antimicrobials for initial empirical therapy, infection treatment, or prophylaxis exhibited significant differences between the groups, along with variations in the average number of prescribed antimicrobials.

**Table 5:** Characteristics and risk factors of matched case-control patients in the point-prevalence study of healthcare-associated infections in adult intensive care units in Brazil

Variables	Total patients	Total matched patients	Matched case	Matched control	Statistical analysis	
	n = 386 (%)	n = 100 (%)	n = 50 (%)	n = 50 (%)	Univariate, P value	Multivariate, P value, OR (95%IC)
Age						
18 64 years	210 (54.4)	57 (57.0)	28 (56.0)	29 (58.0)	1.0000	
≥ 65 years	176 (45.6)	43 (43.0)	22 (44.0)	21 (42.0)		
Length of hospital stay, mean days	14.5	19.6	22.9	16.3	0.0929	
*Underlying conditions*						
Heart disease	106 (27.5)	13 (13.0)	3 (6.0)	10 (20.0)	0.0744	
Systemic arterial hypertension	187 (48.4)	38 (38.0)	21 (42.0)	17 (34.0)	0.5365	
COPD^*^	40 (10.4)	7 (7.0)	4 (8.0)	3 (6.0)	0.7180	
Diabetes	113 (29.3)	31 (31.0)	15 (30.0)	16 (32.0)	1.0000	
Cancer	50 (12.9)	8 (8.0)	7 (14.0)	1 (2.0)	**0.0326**	**0.0446, 13.9559 (1.07-182.80)**
Stroke	52 (13.5)	16 (16.0)	7 (14.0)	9 (18.0)	0.7850	
Kidney disease	52 (13.5)	13 (13.0)	7 (14.0)	6 (12.0)	1.0000	
Liver disease	10 (2.6)	3 (3.0)	2 (4.0)	1 (2.0)	1.0000	
Polytrauma	46 (11.9)	18 (18.0)	9 (18.0)	9 (18.0)	0.7948	
HIV^§^	9 (2.3)	4 (4.0)	1 (2.0)	3 (6.0)	0.6173	
*Hospital and clinical risk factors*						
Surgery	169 (43.8)	42 (42.0)	18 (36.0)	24 (48.0)	0.3110	
Central venous catheter	231 (59.8)	62 (62.0)	34 (68.0)	28 (56.0)	**0.0027**	0.4599, 1.4681 (0.53-4.07)
Peripheral venous catheter	49 (12.7)	7 (7.0)	2 (4.0)	5 (10.0)	0.4360	
Urinary catheter	217 (56.2)	63 (63.0)	36 (72.0)	27 (54.0)	0.0975	
Mechanical ventilation	202 (52.3)	64 (64.0)	39 (78.0)	25 (50.0)	**0.0068**	0.2180, 1.1968 (0.34-4.22)
Tracheostomy	72 (18.6)	27 (27.0)	19 (38.0)	8 (16.0)	**0.0243**	0.7801, 1.1968 (0.34-4.22)
Surgical drain	50 (12.9)	14 (14.0)	5 (10.0)	9 (18.0)	0.3873	
Hemodialysis	34 (8.8)	7 (7.0)	4 (8.0)	3 (6.0)	0.7180	
Enteral nutrition	199 (51.5)	58 (58.0)	35 (70.0)	23 (46.0)	**0.0258**	0.1422, 2.2363 (0.76-6.55)
Parenteral nutrition	8 (2.1)	0	0	0		
*Antimicrobial therapy*						
Use of antimicrobials	231 (59.8)	62 (62.0)	50 (100.0)	12 (24.0)	**< 0.0001**	N/A
Mean of antimicrobials prescribed	1.0	1.1	1.9	0.5	**< 0.0001**	N/A
Empirical use	125 (32.1)	28 (28.0)	28 (56.0)	0	**< 0.0001**	N/A
Infection treatment	55 (14.2)	21 (21.0)	21 (42.0)	0	**< 0.0001**	N/A
Prophylactic use	51 (13.2)	13 (13.0)	1 (2.0)	12 (24.0)	**0.0029**	**0.0071, 0.0296 (0.00-0.38)**

*COPD = chronic obstructive pulmonary disease;

§HIV = human immunodeficiency virus.

According to the logistic regression model presented in **Table 5**, cancer was identified as an independent risk factor for HAIs (odds ratio [OR] = 13.9559; 95% confidence interval [CI] = 1.07-182.80; P = 0.0446), whereas prophylactic use of antibiotics was identified as a protective factor in the control group (OR = 0.0296; 95%CI = 0.00-0.38; P = 0.0071).

## DISCUSSION

The lack of surveillance data and comprehensive prevalence surveys in low- and middle-income countries is concerning.^11^ While several countries are making efforts to quantify the burden and determinants of Healthcare-Associated Infections (HAIs), ensuring data adherence and reliability remains a significant challenge in developing countries.^6,12,13^

In this 24-hour point-prevalence study conducted at 15 participating centers in Brazil, the overall rate of suspected or confirmed HAIs was 26.4% (102/386). This rate exceeded those reported in previous studies conducted in Europe (ranging from 3.0% to 30.7%)^13,14,15,16^ and the United States (ranging from 4.0% to 11.9%).^17,18^ Our results suggest a remarkably high prevalence of HAIs acquired in Brazilian ICUs, regardless of geographic region, consistent with the findings reported by Braga et al.^4^ Although most participating centers were located in the Northern and Southeastern regions, the proportion of patients admitted to the ICU with HAI did not significantly differ across geographic regions.

Considering only the public hospitals evaluated in this study, an extremely high rate of HAIs was observed in adult CSUs (82.5%; data not shown). This finding aligns with previous studies reporting elevated rates of HAIs in Brazilian ICUs.^4,19,20^ Furthermore, due to the COVID-19 pandemic, recent literature has indicated an increase in infection rates associated with healthcare in countries with limited resources, with a reported prevalence increase of up to 15.0%.^21,22^

Another aspect analyzed in this study was the incidence of infection. Our findings confirm that pneumonia (50.0%) and BSI (34.5%) were the most prevalent infections, which is consistent with similar studies conducted in Brazil.^3,4,20,23^ These results reinforce the notion that infections at these anatomical sites are associated with a worse prognosis and higher mortality rates among hospitalized patients in low- and middle-income countries.^24,25,26^ Notably, the etiology of infections, as determined by positive cultures, is an important finding. Our results are consistent with the current literature, with Gram-negative bacilli being the most frequently isolated pathogens in developing countries, including Brazil.^27,28,29^

A concerning aspect to be emphasized in this regard is that only 48.8% of the infectious episodes had microbiological diagnostic criteria, which may contribute to inappropriately high consumption of antimicrobials, either due to the lack of medication de-escalation or the in vitro resistance of microorganisms to the administered antibiotic.^30^ As a result of the absence or delay in microbiological diagnosis, we identified a high rate of initial empirical therapy (54.1%) among the total number of patients using antimicrobials, which are determinant factors for the increase in adverse events in the patient’s clinical course.^31,32^

While multivariate analysis indicated that cancer was an independent risk factor for HAIs, traditional factors such as the use of central venous catheters, mechanical ventilation, tracheostomy, enteral nutrition, and general antimicrobial use were found to be more significant in the group of infected patients.

The excessive use of antimicrobials in Brazil is a healthcare issue that affects all critically hospitalized patients. Studies conducted by researchers in Latin America have reported high prevalence rates of antimicrobial use, particularly of broad-spectrum antibiotics, as we have also demonstrated.^33,34,35,36^ Two primary factors may be associated with this issue: delayed microbiological diagnosis, as previously mentioned, and a lack of diversity in available drugs.^37^ Therefore, the indiscriminate use of antimicrobials is becoming increasingly concerning because it applies selective pressure on microorganisms that progressively restricts the existing therapeutic options through acquired resistance mechanisms.^37,38,39^

Another aspect analyzed, in addition to the previously discussed CSUs, were the findings found in the COUs. Eight ICUs were included, revealing a prevalence of infection of 18.6% (n = 16/86), which is significantly higher than the rates found in developed countries, which typically range from 4.0% to 10.0%.^40,41,42^ Thus, HAIs are a significant complication of cardiovascular procedures, with high morbidity and mortality in affected patients.^40,43^ Similar to data reported in the literature,^40,42,43^ pneumonia was the most common infection in these units.

Acknowledging the limitations of this study in terms of design, time, and resource availability, we believe that the results are well represented and emphasize the importance of conducting similar studies to estimate the burden of HAIs in Brazil. This is particularly relevant given the restricted geographic coverage, data availability, and low participation of centers in this type of surveillance. It is worth noting that such studies can aid in planning and strengthening HAI prevention and control strategies, even in resource-limited settings, particularly for public health in Brazil.

## CONCLUSION

This multicenter study of the prevalence of HAI revealed alarming rates across different regions of the country. Pneumonia and sepsis associated with Gram-negative bacilli are the most significant infections. This group of microorganisms poses a considerable challenge to public health authorities in terms of their content and control. Our findings suggest that the microbiological diagnosis of HAIs falls short of expectations, likely because of the high rates of antibiotic use and empirical treatments.
